# Fibrinogen Level Combined With Platelet Count for Predicting Hemorrhagic Transformation in Acute Ischemic Stroke Patients Treated With Mechanical Thrombectomy

**DOI:** 10.3389/fneur.2021.716020

**Published:** 2021-08-31

**Authors:** Changchun Lin, Hui Pan, Yuan Qiao, Peisheng Huang, Jingjing Su, Jianren Liu

**Affiliations:** Department of Neurology, Shanghai Ninth People's Hospital, Shanghai Jiao Tong University School of Medicine, Shanghai, China

**Keywords:** hemorrhagic transformation, acute ischemic stroke, mechanical thrombectomy, fibrinogen, platelets

## Abstract

A serious complication of acute ischemic stroke (AIS) after mechanical thrombectomy (MT) is hemorrhagic transformation (HT), which is potentially associated with clinical deterioration. This study examined predictors of HT following MT in AIS patients. Patients with AIS due to large artery occlusion in the anterior circulation, treated with MT and successfully recanalized (modified Thrombolysis in Cerebral Infarction score 2b/3), were studied retrospectively. HT was evaluated by computed tomography (CT) 24 h after MT and was diagnosed and classified into parenchymal hematoma (PH) and hemorrhagic infarction (HI). Multivariate logistic regression models were used to determine the risk factors for HT. Receiver operating characteristic (ROC) curve analysis was performed to determine the predictive utility of risk factors for HT. We enrolled 135 patients: 49 in the HT group and 86 in the non-HT group. The two groups differed significantly in baseline fibrinogen levels (*p* = 0.003) and platelet counts (*p* = 0.006). Multivariate logistic regression analyses showed that lower fibrinogen levels [odds ratio (OR), 0.41; 95% CI, 0.23–0.72; *p* = 0.002] and platelet counts (OR, 0.58; 95% CI, 0.33–0.99; *p* = 0.048) were independently associated with a higher risk of HT. Together, the binary variates fibrinogen and platelets well-predicted HT (area under the curve, 0.703; specificity, 77.9%; sensitivity, 55.1%). The combination of fibrinogen <2.165 g/L and platelets <171.5 × 10^9^/L was the strongest predictor of HT (OR, 23.17; 95% CI, 5.75–126.80; *p* < 0.0001). Our study suggests that lower baseline fibrinogen levels and platelet counts may be risk factors for HT in AIS patients following MT and reperfusion. Specifically, the combination of fibrinogen level and platelet count may predict the risk of HT after MT in these patients.

## Introduction

Acute ischemic stroke (AIS) is the leading cause of long-term disability in developed countries and the leading cause of mortality worldwide (1). Mechanical thrombectomy (MT) has become the standard of care for patients with acute intracranial large-vessel occlusion. With the DIFFUSE 3 and DAWN trials extending the time window to up to 24 h, more AIS patients are now eligible for MT (2, 3). Hemorrhagic transformation (HT), a common and severe complication, is usually associated with a poor functional outcome, or even death, after MT and has a reported incidence of up to 46.1% in clinical MT trials (4). Therefore, identifying risk factors for HT could help guide patient selection for MT, which will improve procedural safety and clinical outcomes.

Studies have examined possible risk factors for HT in the setting of MT in AIS patients. Li et al. (5) found that a higher National Institutes of Health Stroke Scale (NIHSS) score, increased systolic blood pressure, history of coronary heart disease, and use of intravenous thrombolysis or oral anti-platelet or anticoagulation drugs were associated with HT in patients undergoing MT. Moreover, ischemic volume, cerebral collateral circulation, baseline Alberta Stroke Program Early CT Score (ASPECTS), and delayed endovascular treatment are associated with an increased risk of HT after MT (6–8). However, most of these risk factors are assessed using clinical and imaging data (9) that are complex and subjective. Hence, it is necessary to identify blood biomarkers that can accurately predict HT after MT.

Studies of blood biomarkers have shown that blood glucose, lipid profiles, bilirubin, aminotransferase, alkaline phosphatase, globulin, biomarkers of disruption of the blood–brain barrier (BBB) (10), inflammation and oxidative stress (11), vasoreactivity (12), and coagulation/fibrinolysis disorder (13–15) are associated with HT in AIS patients (16). These biomarkers may reflect the pathophysiology of HT. However, most of these studies are on thrombolysis treatments, and there are limited data on blood biomarkers and the clinical relevance of HT in the setting of MT.

Platelet and fibrinogen are well-known biomarkers of the coagulation system. Fibrinogen level and platelet counts are proven to be associated with HT in AIS patients after thrombolysis (14, 17, 18). However, research about biomarkers and HT after AIS in the setting of MT is relatively less. Therefore, this study examined blood biomarkers that predict HT in AIS patients after reperfusion to provide reference data facilitating patient selection for MT.

## Materials and Methods

### Study Population

This study recruited 135 AIS patients who had undergone MT and recanalization at the Department of Neurology, Shanghai Ninth People's Hospital, Shanghai Jiao Tong University School of Medicine between October 2012 and May 2018. The inclusion criteria were a diagnosis of AIS confirmed by computed tomography (CT) or diffusion-weighted imaging (DWI), acute anterior circulation occlusion determined by CT angiography (CTA) or digital subtraction angiography (DSA), MT performed within 24 h of symptom onset following reperfusion graded using the modified Thrombolysis in Cerebral Infarction (mTICI) scale (2b/3) with or without intravenous thrombolysis, and routine blood tests before MT and follow-up CT 24 h after MT. Patients were excluded if they had no clinical or laboratory information or follow-up CT imaging.

### Data Collection

Data on sex, age, history of stroke or transient ischemic attack (TIA), and vascular disease risk factors (e.g., smoking, drinking, hypertension, diabetes mellitus, coronary heart disease, and atrial fibrillation) were recorded. Stroke subtypes were based on the Trial of ORG 10172 in Acute Stroke Treatment (TOAST) classification and included large-artery atherosclerosis (LAA), cardioembolism (CE), stroke of other determined cause (ODC), and stroke of undetermined etiology (SUE) (19). Blood pressure was recorded on admission. Neurological deficits were evaluated at baseline, i.e., preoperatively, using the NIHSS. Disability was assessed by the modified Rankin Scale (mRS) score at 90 days after MT. mRS ≤2 was considered a good clinical outcome, while mRS ≥3 was considered a bad clinical outcome.

Imaging parameters included the baseline ASPECTS (20). All patients underwent non-contrast CT and CTA before MT. HT was determined based on 24-h post-interventional non-contrast CT. The CT reading process was performed by two neurologists with more than 2-year working experience in our department. HT was diagnosed and classified into parenchymal hematoma (PH) and hemorrhagic infarction (HI) according to the recommendations of the European Cooperative Acute Stroke Study (ECASS)II classification (21). We had a uniform standard distinction between contrast extravasation after MT and HT. Hyperdensity meeting the following standards was considered to be HT: 1. Hyperdensity with Hounsfield units (HU) <90; 2. Hyperdensity persisting longer than 24 h and/or create mass effect with a hypoattenuation rim; 3. After 24 h, there was still visible hyperdensity on CT (22). Blood samples were obtained before treatments including MT and intravenous thrombolysis and included glucose level, routine blood counts, coagulation function, renal function, electrolytes, and myocardial enzymes. Treatments included intravenous thrombolysis and anticoagulant agents. The procedure time was recorded.

### Endovascular Therapy

Patients were eligible for MT if acute occlusion of the anterior circulation was diagnosed by CTA. Some of the patients within the time window for intravenous thrombolysis, and without contraindications, were given intravenous alteplase as bridging therapy. Patients were treated directly with MT if there was any contraindication to thrombolysis or a heavy thrombus burden. After successful local anesthesia, the patient's femoral artery was punctured to determine the occlusion site. Using a coaxial catheter, the tip of a microcatheter (Rebar™ 21/27; EV3, USA) was placed at the distal end of the occluded artery under micro-guidewire guidance. Solitaire™ AB embolization stents (EV3) 4–6 mm in diameter and 15–30 mm long were selected, according to the diameter of the occluded blood vessels. The stent was introduced into the distal end of the occlusion and then released. Then, contrast agent was injected for visualization, and negative pressure was applied to the catheter to withdraw the stent slowly and remove the thrombus. Recanalization of the main arteries was confirmed by reexamination showing thrombus removal. Evaluation of the grade of recanalization was based on mTICI grade, which is defined as: 0, No perfusion; 1, Minimal flow past the occlusion with little to no perfusion; 2a, Antegrade partial perfusion of less than half of the downstream ischemic territory; 2b, Antegrade partial perfusion of half or greater of the downstream ischemic territory; 3, Antegrade complete perfusion of the downstream ischemic territory. The mTICI scores of 2b/3 were considered recanalization after MT (23).

### Statistical Analyses

Quantitative data were provided as medians and interquartile range (IQR) and categorical variables as frequencies and percentages. Differences in baseline characteristics between the non-HT and HT groups were compared using the Mann–Whitney *U*-test for quantitative data and Pearson chi-square test for categorical variables. Univariate and subsequent multivariate logistic regression analyses were performed to assess the independent risk factors for HT, with adjustment for potential confounders. Potential risk factors of HT including clinical characteristics, vascular disease history, blood biomarkers, intravenous thrombolysis, and treatment time were selected from baseline characteristics as variables in univariate logistic regression analysis. After adjusting for potential confounders, including sex, age, history of smoking and drinking, disease history of hypertension, diabetes mellitus, stroke, coronary artery disease, and atrial fibrillation, all the other factors were selected to perform multivariate logistic regression analysis. Receiver operating characteristic (ROC) curve analyses were performed to investigate the HT prediction efficacy of combinations of binary variates. The cutoff values for binary variates were determined by Youden's index (J) = (Sensitivity + specificity – 1). The point corresponding to the maximum Youden's index was considered a cutoff value. Statistical analyses were performed using SPSS software (ver. 22.0; IBM Corp., Armonk, NY, USA). *p*-values <0.05 were considered statistically significant.

## Results

### Baseline Characteristics and Clinical Outcome of the Study Population

Ultimately, this study enrolled 135 AIS patients treated with MT following reperfusion. HT was diagnosed in 49 patients (36.3%) within 24 h after MT. Table 1 compared the baseline characteristics and clinical outcome of the HT and non-HT groups. The patients with HT had significantly lower fibrinogen levels (*p* = 0.003) and platelet counts (*p* = 0.006), but there were no group differences in the other baseline characteristics and clinical outcome. Table 2 compared the baseline characteristics and clinical outcome of the HI and PH subgroups. Among the 49 HT patients, 29 patients were diagnosed as HI and 20 patients as PH. There was no significant difference in baseline characteristics of the HI and PH groups. But the clinical outcome of the PH group was significantly worse than that of the HI group.

**Table 1:** Baseline characteristics and clinical outcome of the study population.

**Characteristics**	**Non-HT group** ***n*** **= 86**	**HT group** ***n*** **= 49**	***p*** **-value**
Sex (male), *n* (%)	54 (62.8%)	27 (55.1%)	0.488
Age, year, median (IQR)	65.0 (58.0–74.8)	71.0 (62.0–79.0)	0.068
Smoking, *n* (%)	29 (33.7%)	10 (20.4%)	0.149
Drinking, *n* (%)	16 (18.6%)	7 (14.3%)	0.686
Hypertension, *n* (%)	51 (59.3%)	31 (63.3%)	0.787
Diabetes mellitus, *n* (%)	25 (29.1%)	12 (24.5%)	0.709
History of stroke or TIA, *n* (%)	15 (17.4%)	5 (10.2%)	0.375
Coronary artery disease, *n* (%)	11 (12.8%)	10 (20.4%)	0.354
Atrial fibrillation, *n* (%)	35 (40.7%)	22 (44.9%)	0.769
TOAST, *n* (%)			
LAA	52 (60.5%)	27 (55.1%)	0.659
CE	25 (29.1%)	15 (30.6%)	
ODC	1 (1.2%)	0 (0%)	
SUE	8 (9.3%)	7 (14.3%)	
Baseline SBP, mmHg, median (IQR)	150 (132–167)	150 (132–162)	0.788
Baseline DBP, mmHg, median (IQR)	80.0 (71.5–90.0)	80.0 (74.5–95.0)	0.638
Baseline NIHSS, median (IQR)	15 (10–20)	14 (11–17)	0.683
Baseline ASPECTS, median (IQR)	9.0 (8.0–10.0)	8.0 (7.5–9.0)	0.074
Glucose, mmol/L, median (IQR)	8.20 (6.30–9.30)	7.50 (6.53–9.25)	0.342
Leukocyte, × 10^9^/L, median (IQR)	8.62 (6.15–11.20)	8.83 (7.50–10.60)	0.996
Platelet, × 10^9^/L, median (IQR)	207 (177–242)	169 (147–204)	0.006*
Hemoglobin, g/L, median (IQR)	137 (125–148)	137 (131–148)	0.794
PT, s, median (IQR)	11.3 (10.7–11.9)	11.2 (10.5–12.0)	0.866
APTT, s, median (IQR)	25.6 (23.4–27.6)	25.8 (23.8–28.4)	0.427
INR, median (IQR)	0.99 (0.93–1.04)	0.97 (0.92–1.09)	0.952
Fibrinogen, g/L, median (IQR)	2.69 (2.24–3.22)	2.34 (1.88–2.84)	0.003*
D-dimer, mg/L, median (IQR)	0.93 (0.42–2.44)	1.19 (0.51–2.74)	0.286
Creatinine, μmol/L, median (IQR)	73.0 (60.0–89.8)	77.0 (58.0–94.0)	0.682
Urea, mmol/L, median (IQR)	5.05 (4.33–6.38)	5.60 (4.70–7.10)	0.157
Uric acid, μmol/L, median (IQR)	328 (280–417)	321 (241–412)	0.414
Potassium, mmol/L, median (IQR)	3.79 (3.51–4.10)	3.80 (3.54–4.24)	0.605
Sodium, mmol/L, median (IQR)	140 (137–141)	140 (138–141)	0.579
Chloride, mmol/L, median (IQR)	104 (102–106)	104 (101–105)	0.426
Troponin, ng/ml, median (IQR)	0.01 (0–0.03)	0.01 (0.01–0.02)	0.884
BNP, pg/ml, median (IQR)	154 (26–346)	204 (61–342)	0.574
Intravenous thrombolysis, *n* (%)	33 (38.4%)	18 (36.7%)	1
Anticoagulant agent, *n* (%)	16 (18.6%)	13 (26.5%)	0.429
T1, h, median (IQR)	5.54 (4.06–7.56)	5.25 (3.83–7.25)	0.508
T2, h, median (IQR)	7.50 (5.67–9.15)	7.00 (5.58–8.58)	0.570
T2–T1, h, median (IQR)	1.83 (1.25–2.25)	1.50 (1.25–2.50)	0.570
3-month mRS (mRS ≥3), *n* (%)	52 (60.5%)	37 (75.5%)	0.113

*HT, hemorrhagic transformation; IQR, interquartile range; TIA, transient ischemic attack; TOAST, Trial of Org 10172 in Acute Stroke Treatment; LAA, large-artery atherosclerosis; CE, cardioembolism; ODC, stroke of other determined cause; SUE, stroke of undetermined etiology; SBP, systolic blood pressure; DBP, diastolic blood pressure; NIHSS, National Institutes of Health Stroke Scale; ASPECTS, Alberta Stroke Program Early CT Score; PT, prothrombin time; APTT, activated partial thromboplastin time; INR, international normalized ratio; BNP, B-type natriuretic peptide; T1, time from symptom onset to puncture; T2, time from symptom onset to vascular recanalization; T2–T1, time of mechanical thrombectomy treatment; mRS, modified Rankin Scale*.

Bold* values indicate p < 0.05.

**Table 2:** Comparison of the baseline characteristics and clinical outcome according to the subcategorized groups of HT.

**Characteristics**	**HI** ***n*** **= 29**	**PH** ***n*** **= 20**	***p*** **-value**
Sex (male), *n* (%)	19 (65.5%)	8 (40%)	0.141
Age, year, median (IQR)	70.0 (59.0–78.0)	73.5 (62.8–82.5)	0.395
Smoking, *n* (%)	8 (27.6%)	2 (10.0%)	0.254
Drinking, *n* (%)	5 (17.2%)	2 (10.0%)	0.767
Hypertension, *n* (%)	18 (62.1%)	13 (65.0%)	1
Diabetes mellitus, *n* (%)	6 (20.7%)	6 (30.0%)	0.684
History of stroke or TIA, *n* (%)	4 (13.8%)	1 (5.0%)	0.604
Coronary artery disease, *n* (%)	7 (24.1%)	3 (15.0%)	0.675
Atrial fibrillation, *n* (%)	11 (37.9%)	11 (55.0%)	0.374
TOAST, *n* (%)			
LAA	16 (55.2%)	11 (55.0%)	0.992
CE	9 (31.0%)	6 (30.0%)	
ODC	0 (0%)	0 (0%)	
SUE	4 (13.8%)	3 (15.0%)	
Baseline SBP, mmHg, median (IQR)	150 (131–160)	148 (131–170)	0.378
Baseline DBP, mmHg, median (IQR)	87.0 (78.0–95.8)	80.0 (71.0–90.0)	0.428
Baseline NIHSS, median (IQR)	13 (10–17)	14 (13–18)	0.556
Baseline ASPECTS, median (IQR)	10.0 (9.0–10.0)	9.0 (8.0–10.0)	0.122
Glucose, mmol/L, median (IQR)	7.45 (6.53–8.28)	7.95 (6.18–9.48)	0.462
Leukocyte, ×10^9^/L, median (IQR)	8.83 (7.84–10.40)	8.34 (5.48–11.86)	0.394
Platelet, ×10^9^/L, median (IQR)	171 (149–202)	167 (147–205)	0.527
Hemoglobin, g/L, median (IQR)	137 (132–148)	134 (125–150)	0.693
PT, s, median (IQR)	11.5 (10.9–12.1)	11.0 (10.0–12.3)	0.488
APTT, s, median (IQR)	25.8 (23.9–28.8)	25.9 (20.9–27.8)	0.352
INR, median (IQR)	1.00 (0.95–1.08)	0.94 (0.91–1.10)	0.379
Fibrinogen, g/L, median (IQR)	2.29 (1.86–2.78)	2.50 (2.00–2.98)	0.353
D-dimer, mg/L, median (IQR)	0.80 (0.42–2.07)	1.83 (1.02–6.42)	0.521
Creatinine, μmol/L, median (IQR)	84.0 (68.0–102.5)	64.5 (50.5–92.0)	0.394
Urea, mmol/L, median (IQR)	5.50 (4.50–7.00)	6.10 (5.00–7.65)	0.294
Uric acid, μmol/L, median (IQR)	321 (253–396)	323 (221–467)	0.380
Potassium, mmol/L, median (IQR)	3.82 (3.59–4.30)	3.66 (3.39–4.10)	0.527
Sodium, mmol/L, median (IQR)	140 (138–141)	140 (137–143)	0.255
Chloride, mmol/L, median (IQR)	103 (101–105)	105 (103–106)	0.495
Troponin, ng/ml, median (IQR)	0.01 (0–0.03)	0.01 (0.01–0.02)	0.754
BNP, pg/ml, median (IQR)	153 (31–382)	233 (130–301)	0.387
Intravenous thrombolysis, *n* (%)	9 (31.0%)	9 (45.0%)	0.487
Anticoagulant agent, *n* (%)	8 (27.6%)	5 (25.0%)	1
T1, h, median (IQR)	4.38 (3.75–7.15)	5.51 (4.08–7.31)	0.238
T2, h, median (IQR)	6.75 (5.33–8.83)	7.25 (6.21–8.69)	0.442
T2–T1, h, median (IQR)	1.60 (1.25–2.52)	1.33 (1.17–2.24)	0.466
3-month mRS (mRS ≥3), *n* (%)	17 (58.6%)	20 (100%)	0.003*

*HT, hemorrhagic transformation; PH, parenchymal hematoma; HI, hemorrhagic infarction; IQR, interquartile range; TIA, transient ischemic attack; TOAST, Trial of Org 10172 in Acute Stroke Treatment; LAA, large-artery atherosclerosis; CE, cardioembolism; ODC, stroke of other determined cause; SUE, stroke of undetermined etiology; SBP, systolic blood pressure; DBP, diastolic blood pressure; NIHSS, National Institutes of Health Stroke Scale; ASPECTS, Alberta Stroke Program Early CT Score; PT, prothrombin time; APTT, activated partial thromboplastin time; INR, international normalized ratio; BNP, B-type natriuretic peptide; T1, time from symptom onset to puncture; T2, time from symptom onset to vascular recanalization; T2–T1, time of mechanical thrombectomy treatment; mRS, modified Rankin Scale*.

Bold* values indicate p < 0.05.

### Risk Factors for Hemorrhagic Transformation in Acute Ischemic Stroke Patients Treated With Mechanical Thrombectomy

To identify the risk factors for HT in AIS patients after MT, we conducted univariate and multivariate logistic regression analyses (shown in Table 3). After adjusting for potential confounders, including sex, age, and disease history, lower baseline fibrinogen level (OR, 0.41; 95% CI, 0.23–0.72; *p* = 0.002) and platelet count (OR, 0.58; 95% CI, 0.33–0.99; *p* = 0.048) were independently associated with higher odds of HT.

**Table 3:** Univariate and multivariate logistic regression analyses of risk factors for HT in AIS patients with MT.

	**Univariate**		**Multivariate**	
**Parameters**	**cOR (95% CI)**	***p*** **-value**	**aOR (95% CI)**	***p*** **-value**
Sex (male vs. female)	1.38 (0.67–2.80)	0.381		
Age (age in years)	1.76 (1.01–3.05)	0.046		
Smoking	0.50 (0.22–1.15)	0.104		
Drinking	0.73 (0.28–1.92)	0.522		
Hypertension	0.85 (0.41–1.74)	0.650		
Diabetes mellitus	0.79 (0.36–1.76)	0.567		
History of stroke or TIA	0.54 (0.18–1.58)	0.260		
Coronary artery disease	1.75 (0.68–4.47)	0.244		
Atrial fibrillation	1.19 (0.58–2.41)	0.635		
Baseline SBP	0.95 (0.61–1.48)	0.811	0.86 (0.53–1.41)	0.559
Baseline DBP	1.12 (0.70–1.77)	0.637	1.11 (0.68–1.82)	0.671
Baseline NIHSS	0.80 (0.51–1.26)	0.337	0.81 (0.50–1.33)	0.408
Glucose	0.78 (0.51–1.19)	0.244	0.68 (0.40–1.17)	0.163
Leukocyte	1.03 (0.61–1.75)	0.910	1.14 (0.65–2.02)	0.640
Platelet	0.54 (0.32–0.90)	0.017	0.58 (0.33–0.99)	0.048*
Hemoglobin	1.15 (0.73–1.81)	0.538	1.69 (0.92–3.11)	0.094
PT	0.97 (0.86–1.09)	0.570	0.94 (0.75–1.19)	0.620
APTT	1.11 (0.93–1.34)	0.251	1.10 (0.91–1.34)	0.333
INR	1.08 (0.77–1.51)	0.652	1.01 (0.71–1.44)	0.946
Fibrinogen	0.49 (0.29–0.82)	0.007	0.41 (0.23–0.72)	0.002*
D-dimer	1.05 (0.94–1.17)	0.352	1.04 (0.96–1.13)	0.285
Creatinine	1.02 (0.70–1.49)	0.901	1.00 (0.66–1.51)	0.997
Urea	1.16 (0.80–1.68)	0.322	1.16 (0.80–1.68)	0.431
Uric acid	0.83 (0.50–1.39)	0.480	0.83 (0.48–1.45)	0.516
Potassium	1.16 (0.72–1.86)	0.552	1.18 (0.71–1.97)	0.515
Sodium	1.13 (0.79–1.61)	0.493	1.17 (0.81–1.70)	0.407
Chlorine	0.85 (0.57–1.27)	0.418	0.86 (0.56–1.32)	0.494
Troponin	0.94 (0.83–1.07)	0.361	0.90 (0.74–1.09)	0.289
BNP	1.02 (0.72–1.45)	0.908	0.73 (0.44–1.22)	0.229
Anticoagulant agent	1.53 (0.67–3.54)	0.315	1.45 (0.73–2.87)	0.235
Intravenous thrombolysis	0.93 (0.45–1.93)	0.850	1.52 (0.77, 2.98)	0.704
T1, h, median (IQR)	0.92 (0.63, 1.34)	0.676	0.90 (0.60, 1.35)	0.614
T2, h, median (IQR)	0.91 (0.63, 1.3)	0.596	0.89 (0.6, 1.32)	0.550
T2–T1, h, median (IQR)	0.87 (0.52, 1.47)	0.382	0.89 (0.51, 1.56)	0.691

*HT, hemorrhagic transformation; AIS, acute ischemic stroke; MT, mechanical thrombectomy; cOR, crude odds ratio; aOR, adjusted odds ratio; CI, confidence interval; TIA, transient ischemic attack; SBP, systolic blood pressure; DBP, diastolic blood pressure; NIHSS, National Institutes of Health Stroke Scale; PT, prothrombin time; APTT, activated partial thromboplastin time; INR, international normalized ratio; BNP, B-type natriuretic peptide; T1, time from symptom onset to puncture; T2, time from symptom onset to vascular recanalization; T2–T1, time of mechanical thrombectomy treatment*.

Bold* values indicate p < 0.05.

### Predictors of Hemorrhagic Transformation in Acute Ischemic Stroke Patients Treated With Mechanical Thrombectomy

**Figure 1:**
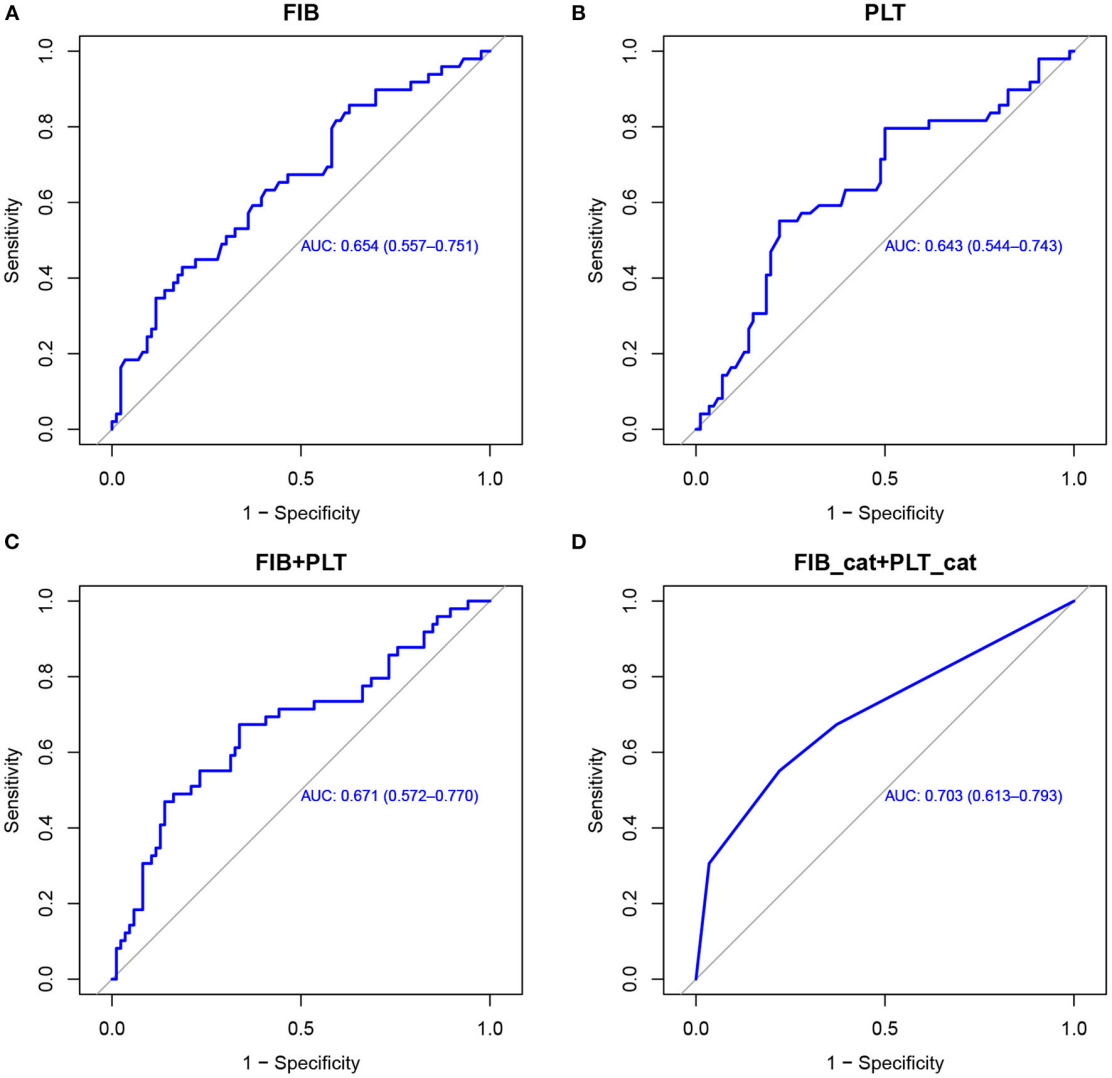
ROC curves used to evaluate the predictive utility of risk factors for HT in AIS patients with MT. Utility of fibrinogen levels **(A)**, platelet counts **(B)**, fibrinogen combined with platelets **(C)**, and a combination of the binary variates fibrinogen (2.165 g/L) and platelets (171.5 × 10^9^/L) **(D)** for predicting HT. ROC, receiver operating characteristic; HT, hemorrhagic transformation; AIS, acute ischemic stroke; MT, mechanical thrombectomy; FIB, fibrinogen; PLT, platelets.

Following logistic regression analysis of HT, we performed ROC curve analysis to evaluate the predictive utility of fibrinogen and platelets. We found that the area under the curve (AUC) for fibrinogen was 0.654 (95% CI, 0.557–0.751), and the cutoff value was 2.165 g/L. The AUC for platelets was 0.643 (95% CI, 0.544–0.743), and the cutoff value was 171.5 × 10^9^/L. The fibrinogen and platelet cutoffs together predicted HT with an AUC of 0.703, specificity of 77.9%, and sensitivity of 55.1%. The positive predictive value was 58.7%, and the negative predictive value was 75.3% (shown in Figure 1).

**Figure 2:**
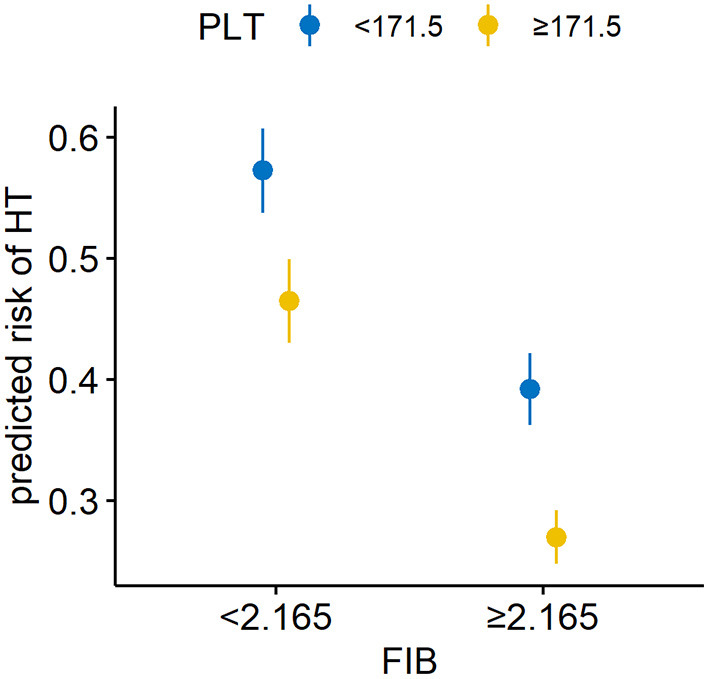
Combined utility of the binary variates fibrinogen and platelets for predicting HT in AIS patients with MT. The combination of a fibrinogen level <2.165 g/L and platelet count <171.5 × 10^9^/L was the strongest predictor of HT. HT, hemorrhagic transformation; AIS, acute ischemic stroke; MT, mechanical thrombectomy; FIB, fibrinogen; PLT, platelets.

Figure 2 and Table 4 showed the predictive power of the binary variates fibrinogen and platelets for HT after MT. Setting fibrinogen ≥2.165 g/L and platelets ≥171.5 × 10^9^/L as references, the combination of fibrinogen ≥2.165 g/L and platelets ≥171.5 × 10^9^/L predicted the lowest risk for HT, while the combination of fibrinogen <2.165 g/L and platelets <171.5 × 10^9^/L was the strongest predictor of HT (OR, 23.17; 95% CI, 5.75–126.80; *p* < 0.0001) (shown in Figure 2; Table 4).

**Table 4:** Combined utility of the binary variates fibrinogen and platelets for predicting HT in AIS patients with MT.

**Parameters**	**Total** ***N***	**HT** ***n*** **(%)**	**OR (95% CI)**	***p*** **-value**
High fibrinogen (≥2.165 g/L)				
High platelet(≥171.5 × 10^9^/L)	70	16 (22.86)	1 (Reference)	-
Low platelet(<171.5 × 10^9^/L)	28	12 (42.86)	2.73 (0.97–7.83)	0.057
Low fibrinogen (<2.165 g/L)				
High platelet(≥171.5 × 10^9^/L)	19	6 (31.58)	1.80 (0.53–5.71)	0.325
Low platelet(<171.5 × 10^9^/L)	18	15 (83.33)	23.17 (5.75–126.80)	<0.0001*

*HT, hemorrhagic transformation; AIS, acute ischemic stroke; MT, mechanical thrombectomy; OR, odds ratio; CI, confidence interval*.

Bold* value indicates p < 0.05.

## Discussion

In 2015, five randomized controlled trials demonstrated the superiority of MT for AIS caused by large-vessel occlusion in the anterior circulation (24). However, the incidence of HT after endovascular treatment varies from 46.0 to 49.5% (4, 8). Based on the radiological appearance, HT is roughly categorized as HI or PH (21). Symptomatic intracerebral hemorrhage (sICH) is defined as a PH2 hematoma with significant clinical deterioration caused by bleeding (25). The rate of sICH in the MT group was 7% in the DIFFUSE 3 trial and 6% in the DAWN trial (2, 3). In our study, HT occurred in 36.3% of the enrolled patients, similar to other studies (4, 8). We roughly categorize HT subtypes into HI and PH. However, we did not differentiate sICH from asymptomatic intracerebral hemorrhage or PH2 from other subtypes of HT since sICH and PH2 may have more clinical implications. Further study should identify more detailed classification to improve clinical utility.

An important finding of this study was the independent effects of decreased fibrinogen levels and platelet counts on hemorrhagic complications in AIS patients who underwent MT following reperfusion. Fibrinogen and platelets are important for hemostatic function, which indicates that there is a relationship between HT and coagulation/fibrinolysis disorder. Bleeding is more likely to occur in conjunction with a coagulation/fibrinolysis disorder; therefore, biomarkers of this system may serve as early predictors of HT incidence in AIS patients with MT (16).

Our study found that lower baseline fibrinogen levels were related to a higher risk of HT after MT. There are limited data on the relationship between fibrinogen and HT in the setting of MT, and most were obtained in the setting of thrombolysis. Wang et al. (17) found that fibrinogen <1.50 g/L was a risk factor for HT after thrombolysis. Conversely, in most studies, a higher baseline fibrinogen level was related to a higher likelihood of HT in AIS patients undergoing thrombolysis (14). Another study found that pre- and post-thrombolysis variation of fibrinogen >200 mg/dl was an independent predictor of sICH (26). Vandelli et al. (27) suggested that a decrease in post-thrombolysis fibrinogen levels of <2 g/L, or of ≥25%, was a risk factor for intracerebral hemorrhage. Yan et al. (28) found that an early decrease in fibrinogen levels was related to sICH after reperfusion therapy with thrombolysis, with or without endovascular thrombectomy. The above studies were based on thrombolysis treatments. Thrombolytic drugs themselves may affect fibrinogen levels (29). Our results indicated that lower baseline fibrinogen levels were associated with a higher risk of HT after MT, which may be attributed to the effect of thrombectomy. Lower preoperative fibrinogen plasma concentration was proven to be associated with excessive bleeding (usually defined as the amount of chest tube drainage after surgery) after cardiac operations in multiple studies (30–32). Fibrinogen is a key protein in the coagulation cascade and thus a potential biomarker for bleeding (33). Under normal pathological conditions, stable blood clot formation is the result of thrombin cleavage of fibrinogen into fibrin. As the amount of insoluble fibrin increases, factor XIII cross-links fibrin monomers forming the matrix for blood clot formation. During the coagulation cascade, fibrinogen concentration is depleted as it is cleaved into fibrin. A lower fibrinogen level may not ensure an appropriate coagulation during and after major surgical procedures (32). To our knowledge, this is the first study of the relationship between preoperative fibrinogen levels and HT in the setting of MT. The patients enrolled in our study underwent MT with or without thrombolysis. Therefore, future study should divide the patients receiving MT into thrombolysis and non-thrombolysis subgroups. It is important to obtain baseline fibrinogen levels and then monitor them during follow-up.

Our study also showed that lower baseline platelet counts were associated with a higher risk of HT after MT. The role of platelet count as a predictor of HT after thrombolysis has been investigated in studies with conflicting results. Some indicated that a lower platelet count does not significantly increase the risk of HT (34, 35). On the contrary, a lower baseline platelet count was suggested to be associated with an increased risk of HT after thrombolysis in another study (18), and clinical guidelines from the American Heart Association/American Stroke Association published in 2018 did not recommend reperfusion therapy in patients with platelets <100,000/mm^3^ (36). However, only a few studies have mentioned HT in the AIS patients treated with MT. Monch et al. (37) were the first ones to reveal that there was no clear association between initial thrombocytopenia (TP), a decline of platelet counts (DPC), and sICH. A recent study also showed no association between pre-procedural platelet count and sICH after thrombectomy (38). Thus, the results of the two studies are contradictory to ours. However, it is difficult to compare these studies due to variations in patient selection, study design, and complicated situation of thrombectomy procedure. Therefore, more research on platelet and HT in the setting of MT will be necessary. Also, considering that platelets have a high turnover during MT, therefore, follow-up of platelet count drop will be needed in future study. Platelets are small blood cells traditionally known for their role in hemostasis. Except for its hemostatic function, platelet also plays an important role in inflammation, angiogenesis, and controlled apoptosis following tissue damage. The term “platelet activation” is used to describe numerous processes, including changes in shape, upregulation of distinct surface molecules, protein synthesis from mRNA and exocytosis, and the release of granule contents. Upregulation of large receptor allows platelets to interact with almost every type of immune cell to mediate immune responses. Activated platelets also secrete a vast range of pro- and anti-inflammatory mediators. Platelets are an abundant source of growth factors, which recruit progenitor cells to the sites of injury to promote angiogenesis, specific regeneration, and tissue remodeling (39). FasL-induced apoptosis has been shown to serve as a controlled way to eliminate inflammation and prevent spreading of inflammatory responses within the eye (40). Blocking of FasL AND platelet depletion led to a decrease in apoptosis of neuronal tissue in models of stroke, suggesting that platelets are an important contributor to the prevention of uncontrolled cell death across tissues including the brain (41). Therefore, we supposed that platelet depletion may play an important role in HT in AIS undergoing MT by decreasing the “platelet activation” process, thus reducing the abilities of hemostasis, inflammatory response, angiogenesis, and tissue repair.

We also derived cutoff points for fibrinogen and platelets, which accurately predicted the risk of HT in AIS after MT. The specificity (77.9%) and sensitivity (55.1%) were best with a cutoff of <2.165 g/L for fibrinogen and <171.5 × 10^9^/L for platelets.

Some limitations of this study must be acknowledged. First, this was a single-center retrospective study with a relatively small sample size, and cause–effect relationships could not be inferred. Multicenter prospective studies are necessary to establish causality and provide more reliable long-term prognostic information. Second, our study roughly categorized HT subtypes into HI and PH; more detailed classification should be applied in future study. Third, due to the effect of fibrinogen depletion, further study is needed to obtain baseline and follow-up fibrinogen data. Fourth, since patients were treated between 2012 and 2018, processes and devices deployed after evidence in favor of MT published in 2015, the patients presumably contained heterogeneity. Therefore, future study will not include patients undergoing MT before 2015. Fifth, the relatively low AUC, specificity, sensitivity, and positive and negative predictive values of fibrinogen and platelet cutoffs together were detected, which may be due to the small sample size. Therefore, larger sample-size studies are needed to validate the result of the present study. Last but not least, we did not conduct further external validation of the model, which leaves this study remaining an exploratory approach on the descriptive level. The patients were enrolled independently from the two hospital districts of our department, which is under independent operation and management from the aspect of doctors, treatments, and laboratory equipment. Therefore, this study was a multicenter analysis theoretically to some extent. However, lacking external validation of the model is one of the greatest limitations of our present study. Therefore, further multicenter prospective research is needed to validate the model in this study.

## Conclusion

Lower baseline fibrinogen levels and platelet counts are associated with HT in AIS patients with anterior circulation large-vessel occlusion after MT. The risk of HT after MT can be predicted by a fibrinogen level <2.165 g/L together with a platelet count of <171.5 × 10^9^/L. Platelet counts and fibrinogen levels as circulatory biomarkers are commonly checked before MT procedure in the emergency room. Therefore, it is feasible that the pre-procedural fibrinogen level and platelet counts may be used as biomarkers to identify patients with an increased risk of HT after MT in AIS patients.

## Data Availability Statement

The original contributions presented in the study are included in the article/supplementary material, further inquiries can be directed to the corresponding authors.

## Ethics Statement

The studies involving human participants were reviewed and approved by the Independent Ethics Committee of Shanghai Ninth People's Hospital. The patients/participants provided their written informed consent to participate in this study.

## Author Contributions

JL conceived the study. JS designed the study and helped revise the manuscript. CL and HP analyzed all data and prepared the drafting of the article. YQ and PH contributed to the acquisition of clinical data. All authors contributed to the article and approved the submitted version.

## Conflict of Interest

The authors declare that the research was conducted in the absence of any commercial or financial relationships that could be construed as a potential conflict of interest.

## Publisher's Note

All claims expressed in this article are solely those of the authors and do not necessarily represent those of their affiliated organizations, or those of the publisher, the editors and the reviewers. Any product that may be evaluated in this article, or claim that may be made by its manufacturer, is not guaranteed or endorsed by the publisher.
